# Expression of Ebolavirus glycoprotein on the target cells enhances viral entry

**DOI:** 10.1186/1743-422X-6-75

**Published:** 2009-06-08

**Authors:** Balaji Manicassamy, Lijun Rong

**Affiliations:** 1 Department of Microbiology and Immunology, College of Medicine, University of Illinois at Chicago, Chicago, Illinois, USA; 2 Department of Microbiology, Mount Sinai School of Medicine, 1 Gustave L Levy Place, Box 1124, New York, New York, USA

## Abstract

**Background:**

Entry of Ebolavirus to the target cells is mediated by the viral glycoprotein GP. The native GP exists as a homotrimer on the virions  and contains two subunits, a surface subunit (GP1) that is involved in receptor binding and a transmembrane subunit (GP2) that mediates the virus-host membrane fusion. Previously we showed that over-expression of GP on the target cells blocks GP-mediated viral entry, which is mostly likely due to receptor interference by GP1.

**Results:**

In this study, using a tetracycline inducible system, we report that low levels of GP expression on the target cells, instead of interfering, specifically enhance GP mediated viral entry. Detailed mapping analysis strongly suggests that the fusion subunit GP2 is primarily responsible for this novel phenomenon, here referred to as *trans *enhancement.

**Conclusion:**

Our data suggests that GP2 mediated *trans *enhancement of virus fusion occurs via a mechanism analogous to eukaryotic membrane fusion processes involving specific *trans *oligomerization and cooperative interaction of fusion mediators. These findings have important implications in our current understanding of virus entry and superinfection interference.

## Background

Enveloped virus fusion with host membrane proceeds via a series of controlled steps which leads to fusion between viral and cellular membranes. The fusion process mediated by class I fusion proteins has been well characterized primarily from our understanding of the pre-fusion and post-fusion structures of influenza haemagglutinin (HA), parainfluenza viruses 3 and 5 F proteins, and HIV glycoproteins [1-9]. First, the receptor-binding subunit binds to its cognate receptor on the host cell surface. Second, the glycoproteins undergo dramatic conformational changes including exposure of the fusion peptide which inserts into the host target membrane, tethering the virions on the host membrane. Third, the fusion protein undergoes additional conformational change forming a coiled-coil structure or six-helix bundle in which the fusion peptide placed apposed to the transmembrane domain. This brings the viral and host membranes to close proximity resulting in the fusion of apposing membranes.

Ebola viral envelope glycoprotein (GP) is involved in mediating virus entry. Ebola GP, like other class I viral fusion proteins, is synthesized as a single polypeptide precursor called pre-GP[10,11]. Pre-GP undergoes modifications by N-glycosylation and O-glycosylation into a fully glycosylated form GP_0_[12,13]. GP_0 _is cleaved in the late-Golgi by furin-like proteases into GP1 and GP2. The newly formed N-terminal end contains the putative fusion peptide[13,14]. On the virion surface GP is present as a trimer and contains two subunits: a surface subunit (GP1) that binds to the cell surface receptor and a transmembrane subunit (GP2) that mediates the virus-host membrane fusion[15]. Previously, we and others have reported that the N-terminal region of GP1 (roughly aa 33-180), referred to as receptor-binding domain or RBD, is involved in receptor binding [16-18]. The GP2 subunit contains an N-terminal putative fusion peptide followed by heptad repeats (N-helix and C-helix, or coiled-coil) which are involved in the formation of the six-helix bundle structure during viral/cell membrane fusion[19,20].

Several cellular factors, such as folate receptor α, DC-SIGN, L-SIGN, hMGL, and Tyro3 family members have been implicated in facilitating Ebola entry [21-26], but the primary receptor has not been identified yet. Based on the current understanding of Ebolavirus entry, Ebola infection is believed to be initiated by binding of GP1 to the cellular receptor(s). Once bound, the virus is endocytosed into the endosomes. In the endosomes, under low pH, GP is cleaved by endosomal cysteine proteases such as cathepsin B and cathepsin L[27,28]. Specific inhibition of this cleavage event drastically affects virus entry[27]. It has been speculated that this cleavage event under low pH might acts as a trigger for GP2-mediated viral/cell membrane fusion. At the end of virus-host membrane fusion, the viral capsid is released into the cytoplasm and virus replication takes place.

Although Ebola and Marburg virus GP1 subunits share ~ 35% overall sequence conservation [29], we and others have shown that Ebola and Marburg viruses are likely to share a common receptor or co-factor in viral entry[16,17]. Another piece of evidence is that transient overexpression of Ebola GP or Marburg GP in the host cells can specifically block both EGP/and MGP/HIV pseudovirus entry[30]. This is consistent with the observations that viral infections can render cells to become resistant to re-infection by the same virus or viruses using the same receptor. It is thought that in these cells, the newly synthesized glycoprotein forms a complex with the viral receptor and hence, there are fewer free viral receptors available for re-infection. This phenomenon of superinfection resistance or receptor interference has been observed among the retroviruses using the same receptor[31].

To further characterize the mechanism of Ebolavirus entry, in this study we have generated stable cells expressing EGP under a tetracycline inducible promoter. This system allows us to regulate EGP expression by modulating the concentration of inducer (doxycycline). Surprisingly, EGP expression in target cells specifically enhanced EGP/HIV pseudotyped virus transduction. We have systematically mapped and characterized the functional domains in EGP involved in this *trans *enhancement. Our results show that the fusion machinery, but not the receptor-binding, of Ebola GP, is responsible for this enhancement. Therefore, EGP expression in target cells displays a dichotomous property: it can specifically block EGP-mediated viral entry, which is mediated by the receptor-binding region of GP1, or it can specifically enhance Ebola GP-mediated viral entry via the fusion machinery of GP, mostly by GP2. Based on these results, we propose a model for EGP mediated *trans *enhancement, which mirrors the current models of intracellular membrane fusion and cell-cell fusion. Furthermore, this study may have important implications on our understanding of virus entry and superinfection interference.

## Results

### Expression of Ebola glycoprotein in HEK cells enhances transduction of EGP/HIV pseudovirions

We previously demonstrated that transient transfection and over-expression of Ebola glycoprotein on target cells can specifically inhibit entry of EGP/HIV or Marburg glycoprotein (MGP)/HIV pseudovirus (Additional file 3, Figure S1; [30]). To better characterize the entry mechanism of these viruses, a HEK (293) cell line stably expressing the full length EGP under a tetracycline inducible promoter (EGP Tet-On) was generated. This system allows us to modulate EGP expression by varying the concentration of doxycycline (dox; an analog of tetracycline).

**Figure 1:**
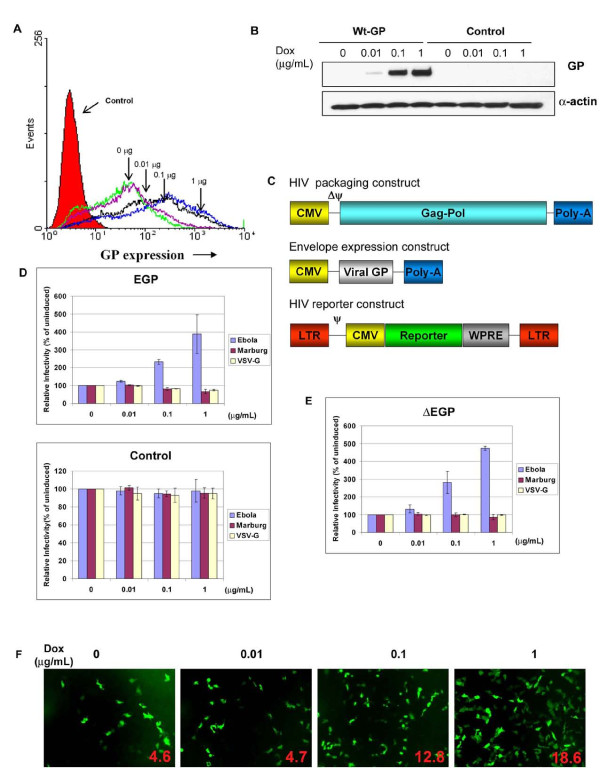
**EGP expression in target cells enhances EGP/HIV transduction**. (A) Cell surface expression of EGP. EGP Tet-On cells were seeded in 12-well plates (6 × 10^4 ^cells/well) and EGP expression was induced with indicated concentrations of dox. After 24 h post-induction, cell surface EGP levels were analyzed by flow cytometry using an EGP monoclonal antibody. (B) Western blot analysis of EGP expression in Tet-On cells. EGP Tet-On cells were seeded in 12-well plates and induced with indicated concentrations of dox. Forty-eight hours post-induction, cell lysates were subjected to SDS-PAGE followed by immunoblotting using a EGP monoclonal antibody. (C) HIV pseudotyping constructs. HIV packaging construct encodes gag/pol genes required for virion assembly. Envelope expression construct encodes genes for EGP or MGP or VSV-G under the control of a CMV promoter. HIV reporter construct encodes the viral genomic RNA, carrying a luciferase or a GFP reporter gene. (D) Enhancement of EGP/HIV transduction by EGP expression in target cells. EGP or control Tet-On cells were seeded in 24-well plates (3 × 10^4 ^cells/well) and induced with varying concentrations of dox. After 24 h post-induction, cells were challenged with EGP/HIV, MGP/HIV or VSV-G/HIV pseudovirions carrying a luciferase reporter gene. The luciferase activities in the cell lysates were measured 48 h post-infection and are presented as percentage of the uninduced cells (100%). Data represents an average of at least three independent experiments. Bars, standard deviations. (E) The mucin-like region in EGP is not required for enhancement. Tet-On stable cells with EGP mutant lacking the mucin-like region (ΔEGP) were induced with dox and challenged with pseudotyped virions carrying luciferase reporter. The luciferase activities are shown as relative percentage of the uninduced cells (100%). Data represents an average of at least three independent experiments. Bars, standard deviations. (F) EGP Tet-On cells infected with EGP/HIV pseudovirions carrying a GFP reporter. The percentage of GFP expressing cells, shown in each panel as inserts, were quantified by flow cytometry.

To examine the EGP induction profile, the EGP Tet-On cells were incubated with increasing concentrations of dox (0, 0.01, 0.1, 1 μg/ml), and the EGP surface expression levels were measured by flow cytometry. Surface staining of these cells with a monoclonal anti-EGP antibody showed a dose-dependent increase in surface EGP levels as the concentration of dox increased (Figure 1A). Compared to the control Tet-On cells (empty vector, red), the uninduced EGP Tet-On cells showed a clear shift in mean fluorescent intensity (green), suggesting a low level of leaky EGP expression without induction. Nevertheless, increasing mean fluorescent intensities were detected as the concentration of dox was increased, indicating that EGP expression was induced by dox. The levels of surface EGP in the Tet-inducible system, even at 10 μg/ml, was approximately 10-fold lower than the transient over-expression in 293T cells (data not shown). Western blot analysis of the EGP Tet-on cells confirmed the dose-dependent induction of EGP expression (Figure 1B). However, EGP expression was not detectable in the uninduced cells, likely due to lower sensitivity of Western blot analysis than flow cytometry. In addition, we did not observe any visible cytopathic effects of EGP expression on the EGP Tet-On cells.

To examine the effect of EGP expression on the target cell on EGP/HIV pseudovirus infection, the EGP Tet-On cells were induced with increasing concentrations of dox (0, 0.01, 0.1, 1 μg/ml) for 24 hrs and challenged with the EGP/HIV pseudovirions which were generated from the producer cells (293T) by transient transfection of three plasmids (Figure 1C). Forty-eight hours post-infection, the luciferase activities in the infected cells were determined as a measure of the pseudotyped virus transduction, since the HIV vector carries a luciferase reporter gene. Surprisingly, expression of EGP in the target cells enhanced, instead of blocking, the transduction of the EGP/HIV pseudovirions in a dox dose-dependent manner (Figure 1D, top). The luciferase activities in the target cells (expressed as relative infectivity, % of uninduced cells) increased correspondingly as increasing concentrations of dox were used, reaching up to 4-fold at 1 μg/ml of dox. In contrast, neither MGP nor VSV-G- mediated transduction was enhanced in these cells with dox induction (Figure 1D, top). Further, treatment of the control cells (empty vector) with dox did not affect EGP- or MGP- or VSV-G- mediated viral transduction (Figure 1D, bottom).

To further confirm and quantify the observed *trans *enhancement by EGP, the dox induced EGP Tet-On cells were challenged with the EGP/HIV pseudovirions carrying a green fluorescent protein (GFP) reporter instead of luciferase, and the GFP-positive cells were visualized by fluorescent microscopy (Figure 1F) and quantified by flow cytometry (Figure 1F, inserts). Increasing number of GFP-positive cells was detected as higher concentrations of dox were used for induction. The GFP-positive cells were about 4-fold higher at 1 μg/ml of dox (18.4%) than the uninduced cells (4.6%), very consistent with the results described above using the EGP/HIV virus carrying the luciferase gene. These results demonstrate that EGP expression in the target cells can specifically enhance, rather than blocking, the EGP-mediated transduction.

### The mucin-like region of GP1 is not required for *trans *enhancement of EGP-mediated transduction

The C-terminus of Ebola GP1 contains a mucin-like region (~ 200 residues in length) which is heavily glycosylated and is not required for viral entry [12,16]. To examine whether the mucin-like region was involved in enhancement, an HEK Tet-On cell line expressing ΔEGP (Δ309-489), which lacks the mucin-like region, was generated. The induction profiles of the ΔEGP in this cell line, examined by flow cytometry, were comparable to that of the Wt EGP (Additional file 4, Figure S2). Furthermore, like Wt EGP Tet-On cells, the ΔEGP Tet-On cells could specifically enhance the EGP-mediated, but not MGP- or VSV-G -mediated transduction in a dose dependent manner, up to approximately 5-fold higher at a dox concentration of 1 μg/ml than the uninduced cells (Figure 1E). These results indicate that the mucin-like region of EGP is not involved in *trans *enhancement of the EGP-mediated transduction.

### *Trans *enhancement by EGP is correlated with the entry susceptibility of the target cells

To test whether the observed *trans *enhancement of EGP/HIV transduction was dependent on the entry susceptibility of the target cells, the wt EGP Tet-On and the ΔEGP Tet-On cell lines were derived from a susceptible cell line (HeLa) or a resistant cell line (Human T lymphocytes -Jurkat). Expression of wt EGP and ΔEGP in these cells were examined by both western blot analysis and flow cytometry, and a dose dependent induction by dox was observed (data not shown).

**Figure 2:**
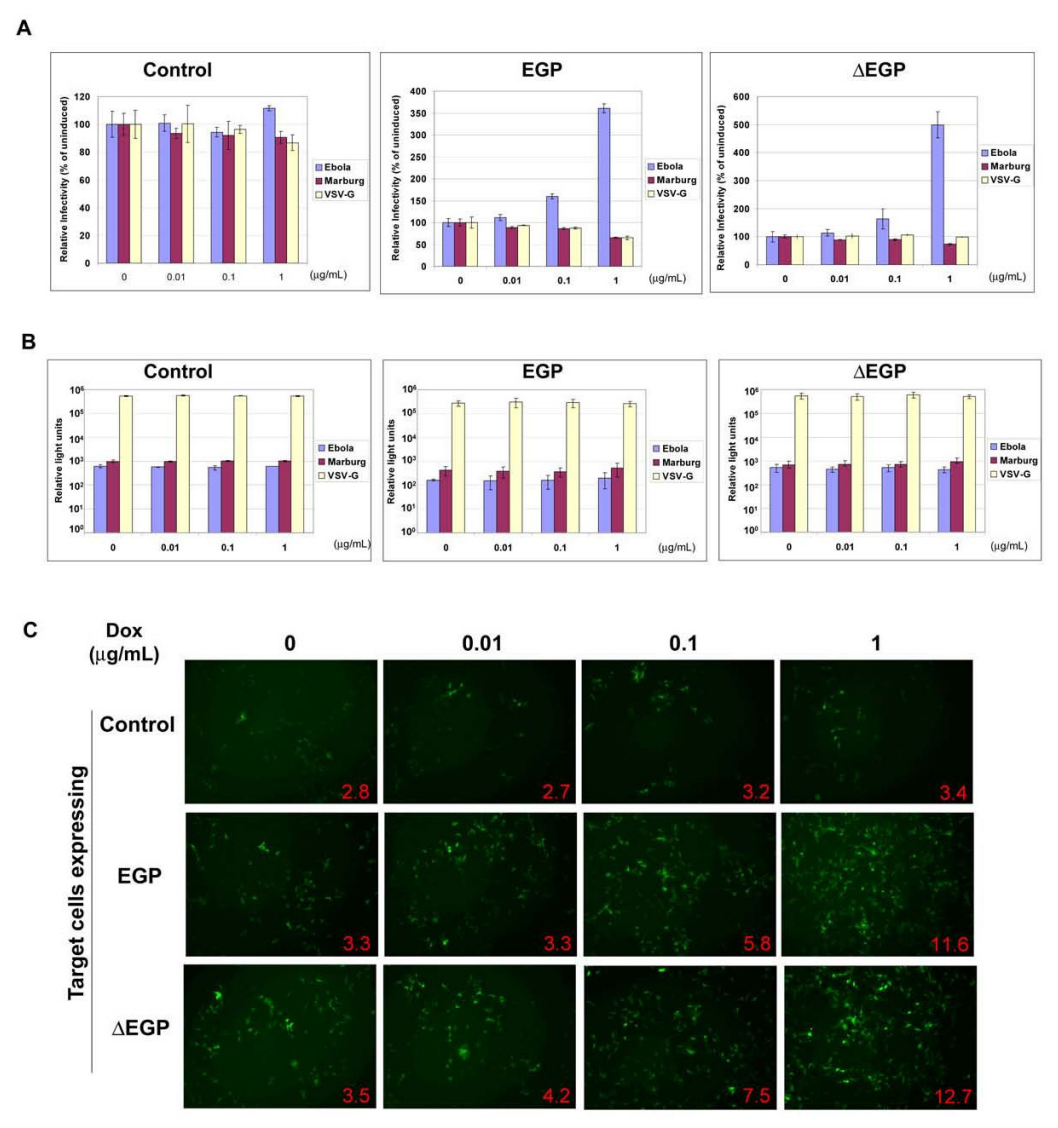
**GP enhancement is correlated with the entry susceptibility of the target cells**. (A) EGP expression in HeLa cells enhances EGP/HIV transduction. HeLa Tet-On cells with EGP, ΔEGP or control vector were induced with dox and challenged with pseudotyped viruses. The luciferase activities were measured 48 h post-infection and are shown as percentage of the uninduced cells (100%). Data represents an average of at least three independent experiments. Bars, standard deviations. (B) EGP expression in Jurkat Tet-On cells does not enhance EGP/HIV transduction. Jurkat Tet-On cells expressing EGP or ΔEGP  or control vector were challenged with luciferase reporter virus and luciferase activity in the cell lysates are shown in relative light units (RLU). Data represents an average of at least three independent experiments. Bars, standard deviations. (C) Enhancement of EGP/MLV pseudovirus transduction in HeLa Tet-On cells. HEK Tet-On cells with EGP, ΔEGP or control vector induced with dox were challenged with ΔEGP/MLV pseudovirion carrying a GFP reporter. The percentage of GFP expressing cells are shown as inserts in each panel.

Challenging the HeLa cells bearing either wt EGP or ΔEGP with EGP/HIV pseudovirus led to a dose-dependent enhancement of transduction (Figure 2A), consistent with the results in the HEK cells described above. In stark contrast, expression of either wt EGP or ΔEGP in Jurkat cells did not enhance the transduction efficiency of the pseudovirus (Figure 2B). Together these results suggest that (receptor) binding of the GP/HIV virions to the target cells is important for EGP-mediated enhancement.

### EGP expression on the target cells enhances EGP/MLV pseudovirion transduction

To confirm that enhancement by EGP expression on target cells is independent of the HIV pseudotyping system, HEK Tet-On cells expressing either EGP or ΔEGP induced with dox were challenged with murine leukemia virus (MLV)-based pseudvirions (EGP/MLV) carrying a GFP reporter. It is clear that number of GFP-positive cells increased in a dox dose-dependent manner in HEK cells expressing either EGP or ΔEGP to levels similar to that observed with the EGP/HIV pseudovirions (~ 3.5- 4 fold). In contrast, EGP/MLV transduction was not affected with dox treatment in control cells (Figure 2C). In addition, MGP/MLV or VSVG/MLV-mediated transduction was not affected in EGP or ΔEGP-expressing cells (data not shown). These results demonstrate that EGP-mediated *trans *enhancement of viral transduction is specific to EGP-bearing virions and independent of the pseudotyping system used for viral entry.

### *Trans *enhancement is more pronounced for coiled-coil mutants of GP2

**Figure 3:**
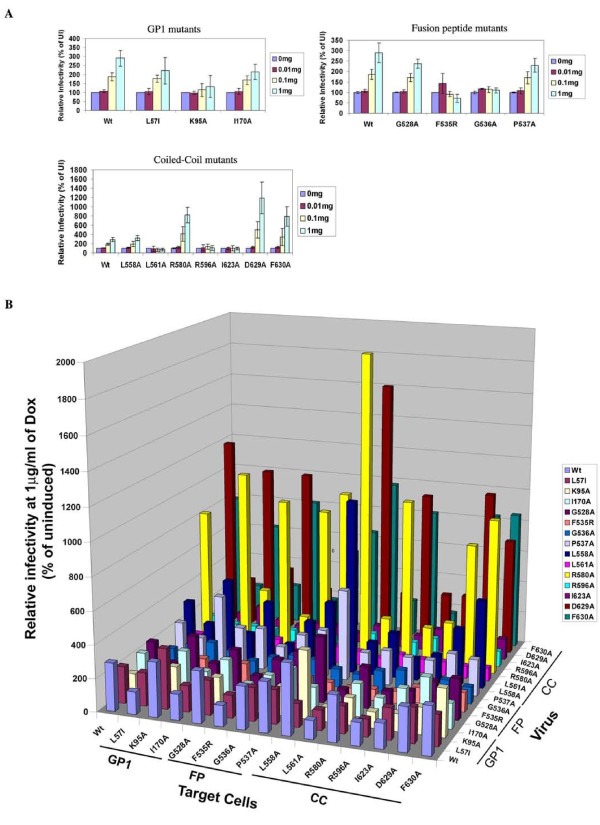
**Analysis of Ebola GP mutants in *trans *enhancement process**. (A) HEK Tet-On cells expressing Wt EGP were challenged with EGP mutant viruses, GP1 mutants, relative infectivity of Wt and GP1 mutant viruses. Fusion peptide mutants, relative infectivity of Wt and fusion peptide mutant viruses. Coiled-coil mutants, relative infectivity of Wt and GP2 mutant viruses. (B) Matrix analysis of *trans *enhancement by EGP. Wt or mutant EGP expressing HEK Tet-On was either uninduced or induced with 1 μg/ml of Dox. After 24 h, the cells challenged with pseudovirus particles carrying Wt or mutant EGP. The luciferase activities in infected cells are represented as relative percentage of luciferase activity in uninduced cells. Data represents an average of three independent experiments. For clarity of the chart we have omitted the error bars in panel B.

To further understand the mechanism of *trans *enhancement, HEK Tet-On cells expressing Wt EGP were challenged with the EGP/HIV pseudotyped viruses with mutation in different functional regions of EGP namely the receptor-binding domain (RBD), fusion peptide and coiled-coil region (Figure 3A). These EGP mutants encompassing different functional domains of the glycoprotein have been previsouly described to be similar to Wt EGP in protein expression and incorporation pseudoparticles (Additional file 6, Figure S4). However, they have distinct defects in different steps of viral entry (Table 1) [16]. It is apparent from Figure 3A that Wt EGP on the target cells significantly enhanced transduction of several coiled-coil mutant EGP/HIV pseudoparticles. Especially, mutants R580A, D629A, and F630A showed enhancement in infectivity nearly 824%, 1,188%, and 789%, respectively. Also, some of the RBD and fusion peptide mutants showed enhancements similar to Wt EGP/HIV particles (200%). However, expression of Wt EGP in target cells did not significantly enhance the infectivity of mutant EGP/HIV pseudoparticles that were severely defective in mediating viral entry (Infectivity less than 0.1% of Wt; Table 1). An exception to this was RBD mutant K95A, a receptor-binding mutant, whose transduction was not enhanced by Wt EGP. However, we observed enhancement for RBD mutants L57I and I170A, which are defective in post- receptor binding steps of entry (J Wang, BM and LR, Manuscript in preparation).

**Table 1:** Comparison of relative infectivity and enhancements for different EGP mutants

**Mutant**	**Relative Infectivity in 293T Cells (%)**	**Relative Enhancement in Wt EGP Tet-On cells(%)**
Wt	100	290
L57I	9.50	221
K95A	4.00	133
I170A	26.10	213
G528A	74.42	238
**F535R**	**0.02**	**71**
**G536A**	**0.07**	**109**
P537A	3.37	230
L558A	6.92	324
**L561A**	**0.02**	**73**
R580A	7.80	824
**R596A**	**0.02**	**108**
**I623A**	**0.02**	**96**
D629A	0.74	1188
F630A	0.68	789

*Mutant completely defective in mediating viral entry are shown in bold font

To thoroughly characterize this trans enhancement phenomenon, HEK Tet-On cells expressing fourteen EGP mutants were generated (3 in GP1, 5 in fusion peptide, and 6 in coiled-coil region; Additional file 4, Figure S2 and Additional file 5, Figure S3). HEK cell lines expressing a specific mutant EGP or wt EGP (induced with 0, or 1 μg/ml of dox) were challenged with each of the fifteen different types of HIV pseudovirions bearing either mutant or wt EGP, and the luciferase activities of each infected cell line were determined and the results plotted in Figure 3B. Several interesting conclusions can be drawn from the data:

(1) No complementation was observed between severely defective mutants. The most impaired GP mutants expressed in target cells could not *trans *enhance viral entry mediated by GP mutations at other positions (same region or different regions) or themselves. In addition, at least partially functional EGP is required on both target cell and on the virions for *trans *enhancement. For example, mutants F535R and L561A are completely impaired in mediating viral entry, and thus they were unable to *trans *enhance either any other mutant or themselves.

(2) *Trans *enhancement is cooperative. A partially impaired GP on the target cells could nevertheless *tran*s enhance viral entry mediated by the same defective GP. For example, Expression of L558A on the target cells was as good as wt GP in *trans *enhancing L558A-mediated viral entry. This phenomenon is also true for mutants R580A, D629A, and F630A, and other mutants. This is quite remarkable considering that these mutants are fairly impaired in their ability to mediate viral entry. For example, it was shown that F630A displayed less than 1% of wt GP activity in mediating viral entry (see Table 1), but it could *trans *enhance just like wt GP (Figure 3B). The cooperative (but not complementary) feature of *trans *enhancement provides a nice explanation for specificity: Ebola GP on the target cell can only enhance Ebola GP, but not Marburg GP-mediated viral entry (see Discussion).

(3) As described above, higher enhancement indices (EIs) were observed for coiled-coil region mutants. For example, mutant L558A on the target cells could increase R580A-mediated viral entry by almost 19 folds (EI = 1894%). These results further strengthen the hypothesis that the fusion function, rather than the receptor-binding function, of GP is involved in *trans *enhancement.

### Entry enhancement of Ebolavirus-like particles by EGP expression

**Figure 4:**
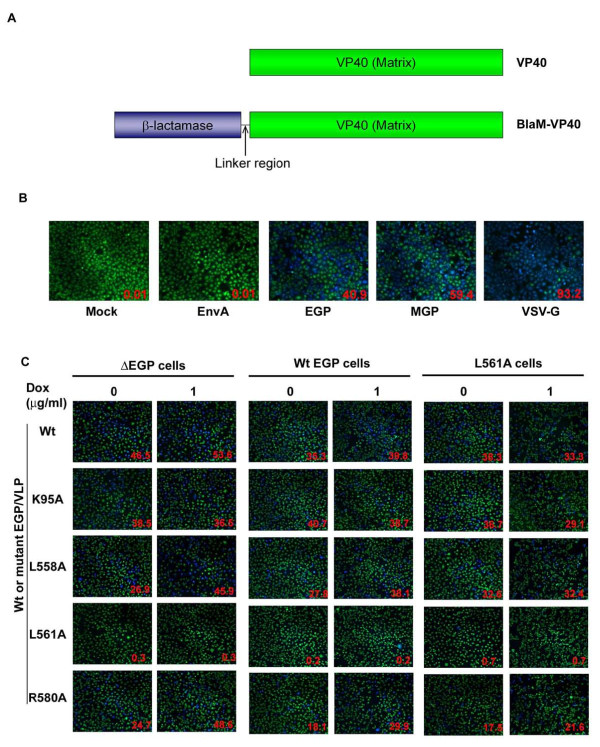
**β-lactamase based Ebola VLP fusion assay**. (A) Schematic representation of VP40 and BlaM-VP40 chimera. (B) BlaM based VLP fusion assay. HEK cells were infected by spinoculating at 1,500 rpm for 2 h (4°C) followed by incubation at 37°C for 3 h. The cells were loaded with CCF4-AM dye and analyzed by fluorescence microscopy. The percentage of infected cells were quantified by flow cytometry and shown as inserts. (C) BlaM VLP fusion assay in EGP expressing cells. EGP expressing cells challenged with Wt or mutant EGP carrying BlaM-VLP. The cells were loaded with CCF4-AM dye and analyzed by fluorescence microscopy. The percentage of infected cells were quantified by flow cytometry and shown as inserts.

The entry pathways of EGP/HIV and EGP/MLV pseudoviruses are generally believed to faithfully mimic the Ebolavirus entry. However, Ebolavirus particles are filamentous, pleomorphic and morphologically distinct from retrovirus particles which are usually spherical. Previously, it has been shown that expression of EGP and the matrix protein VP40 in cells results in production of virus-like particles (VLPs), and these VLPs are morphologically similar to Ebolavirus particles[32]. Thus we have developed a modified β-lactamase based VLP entry assay to further investigate the EGP-mediated *trans *enhancement [24]. Modified β-lactamase protein was fused to the N-terminus of VP40 (Figure 4A) to create BlaM-VP40. Upon VLP fusion the viral matrix will be released into the cytoplasm and the β-lactamase activity in these cells can be used as a measure for the GP-mediated viral entry.

To examine if BlaM-VP40 could be incorporated into VLP, 293T cells were transfected with BlaM-VP40 and EGP expressing plasmids. After 48 h, the supernatants were collected and analyzed for BlaM-VP40 incorporation by western blot. BlaM-VP40 was efficiently incorporated in the VLPs (data not shown). However, the incorporation levels were slightly lower than the unmodified VP40. We next examined if the β-lactamase activity could be detected in target cells upon incubation with the VLPs. HEK cells were infected with EnvA/, EGP/, MGP/, or VSVG/VLP by spinoculation at 4°C followed by incubation at 37°C. After 3 hour incubation at 37°C, the cells were stained with β-lactamase cleavable dye CCF2-AM or CCF4-AM for 1 h and immediately visualized under a confocal microscope (Figure 4B). Mock and EnvA/VLP incubated HEK cells (non-permissive for EnvA-VLP) were completely negative for β-lactamase activity (green), because EnvA, the glycoprotein of avian leucosis and sarcoma virus A, requires the cognate receptor Tva (which is absent in HEK cells) for viral entry [33,34]. In HEK cells infected with VSVG/VLPs, nearly 93% of the cells were β-lactamase postive (blue). In EGP/or MGP/VLP challenged HEK, 41% and 50% cells were positive for β-lactamase activity, respectively. These results indicate that this VLP system recapitulates the fusion/entry properties of the respective viral glycoproteins. Therefore, we chose to use this system as a safe and surrogate assay to further investigate the EGP-mediated *trans *enhancement.

We next examined if EGP expression in target cells could enhance fusion/entry of VLPs. Stable HEK Tet-On cells expressing ΔEGP or EGP or mutant L561A were induced with 0 or 1 μg/ml of dox and challenged with wt/or mutant EGP/VLPs. In ΔEGP or Wt EGP expressing cells, only a slight increase in number of blue cells (fused with VLPs) was consistently observed for wt EGP/VLPs, giving 15% or 12% more blue cells under induced than uninduced conditions, respectively (Figure 4C). More significantly, approximately 1.5-2 fold increase in blue cells was observed for mutants L558A/VLPs and R580A/VLPs, demonstrating *trans *enhancement on the VLPs. In contrast, expression of ΔEGP or EGP in the target cells did not affect fusion/entry of mutants K95A/VLPs, consistent with the results using HIV pseudovirions (Figure 4C and 3A). These results further substantiate our notion that, like GP/HIV pseudovirions, fusion/entry of GP/VLPs requires efficient binding of the VLPs to the target cells. Also consistent with the results of GP/HIV pseudovirions, the L561A-expressing HEK cells did not enhance fusion/entry of wt or mutant GP/VLPs.

It is important to point out that although the general trend for *trans *enhancement is similar for both GP/HIV pseudovirons and GP/VLPs, there are some distinct differences for different mutants in mediating fusion/entry of VLPs compared to that of HIV pseudovirions. For example, mutant L561A, which is completely impaired in mediating viral entry measured by HIV pseudovirion entry assay (0.02% of wt GP, see Table 1), is also defective using VLPs (Figure 4C). However, mutants K95A, L558A and R580A, which have lower levels of relative infectivity in the HIV-based entry assay (<8% of wt EGP), had higher levels of relative infectivity in the VLP based assay (>50% of wt EGP). Further investigation is needed to elucidate these differences. Also, the observed differences in *trans *enhancement using HIV pseudovirion-based and VLP-based assays need to be further characterized in the future. Nevertheless, our results suggest that EGP expression in target cells can enhance Ebolavirus entry.

## Discussion

### A molecular model for *trans *enhancement by EGP

**Figure 5:**
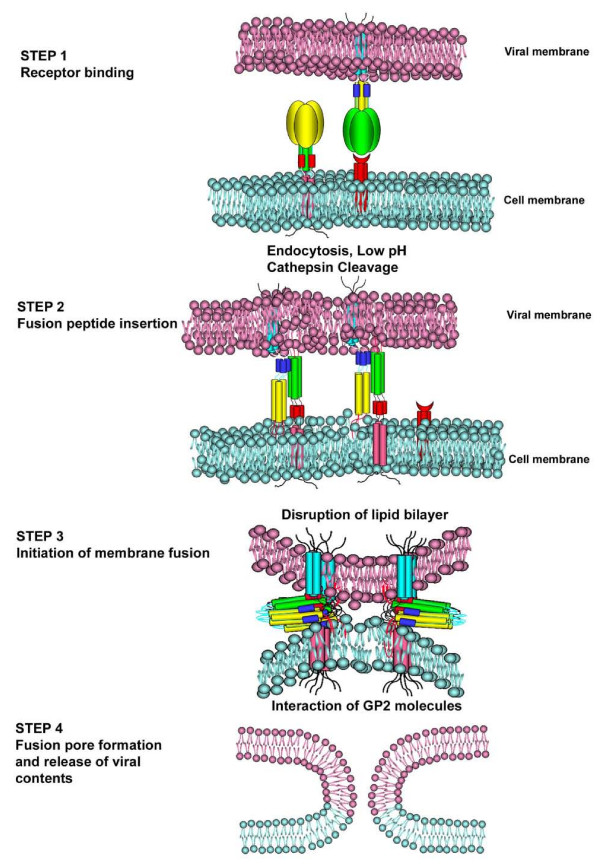
**A proposed model for EGP-mediated enhancement**. Step 1. Receptor binding. EGP binds to its cell surface receptor(s). The bound virus is endocytosed into the cell, where it undergoes cleavage by cysteine proteases (cathepsin B and L) under low pH environment. Step 2. Fusion peptide insertion. The fusion peptide from the virion EGP is inserted into the host membrane. Also, EGP of the target cell inserts to the viral membrane through the fusion peptide. Step 3. Initiation of membrane fusion. Conformational changes occur on EGPs and lead to direct contact and interaction between the viral membrane-anchored EGP and the cell membrane-anchored EGP, forming an oligomeric complex. Membrane fusion ensues. Step 4. Membrane fusion. Fusion pore forms and host-viral contents mix.

The results presented in the current study demonstrate that expression of EGP on the target cells can enhance EGP-mediated viral entry. Here we propose a molecular model based on our current understanding of this novel phenomenon (Figure 5). Ebolavirus binds to the target cells via interactions of the RBD of Ebola GP1 and the cellular receptor(s), and the attached virions are endocytosed into the endosomes (step 1). In the endosome, both the cell- and viral-anchored EGPs are cleaved by proteases cathepsin B/L, triggering a series of conformational changes on EGP which are essential for EGP-mediated membrane fusion. One of the required structural changes is to expose the fusion peptide of GP2, which is buried internally in the native, prefusion state of EGP trimer, and insert the fusion peptides to the opposing membrane (step 2). Concurrently or subsequently, the trimeric GP2 molecules on the virion (either in a pre- or post-six helical bundle form) "kiss" and "engage" the cell-anchored trimeric GP2 molecules, leading to formation of higher order GP2 oligomers consisting of at least two (likely multiple) GP2 trimers, with at least one trimer from the cell-anchored GP2, initiating membrane fusion (step 3). Thus, bidirectional interaction of EGP enhances the later stages of fusion process such as hemifusion, fusion pore formation (step 4).

There are three important features in the current model which distinguish *trans *enhancement from the conventional view on EGP-mediated viral entry. First, geometrically, *trans *enhancement is coordinated by both viral- and host-anchored EGPs instead of the unidirectional mediation by the viral-anchored EGP alone. This bidirectional geometry of EGP-mediated viral entry is reminiscent of intracellular vesicle transport where the v-SNARE is carried on the transport vesicle and the t-SNARE on the target membrane, and the bidirectional v- and t-SNARE interaction mediates membrane fusion [35]. Also, EGP mediated fusion enhancement is analogous to the cell-cell fusion mediated by *Caenorhabditis elegans *proteins AFF-1 and EFF-1 through the formation of *trans *homotypic oligomers [36,37]. The synergistic (or additive) effect of the GP2 molecules on the opposing membranes (bidirectional) may be more efficient than the GP2 on the viral membrane alone (unidirectional) in mediating viral/cell membrane fusion.

Second, physically, we propose that the fusion machinery, but not the receptor-binding function of the cell-anchored EGP is responsible for *trans *enhancement. This is in contrast to the role of the viral-anchored EGP in viral entry where both receptor-binding and fusion functions of EGP are required. The role of the fusion machinery of EGP in *trans *enhancement is demonstrated using a series of EGP mutants in this study. For example, mutant K95A, which is defective in mediating viral entry (4% of wt EGP) due to the impaired receptor-binding, could still *trans *enhance at a level similar to that of wt EGP. In contrast, two putative post-receptor binding mutants (L57I and I170A) displayed more impaired phenotype in *trans *enhancement (see Figure 3B). Based on our binding data and structure of EGP, it is clear that these residues are critical for triggering the conformational changes on EGP which are critical for EGP-mediated membrane fusion. Further, several substitution mutations either in the fusion peptide or coiled-coil of GP2, the fusion subunit of EGP, were completely defective in facilitating *trans *enhancement (see Figures 3B). In addition, the most pronounced enhancement by EGP was on the mutants in the coiled-coil region (see Figure 3A). Together, these results strongly suggest that GP2, but not the receptor-binding function of GP1, is responsible for *trans *enhancement.

Third, mechanistically, we hypothesize that the cell-anchored and viral-anchored EGPs interact and form higher order oligomeric structures, analogous to the *trans*-SNARE complexed formed by v- and t-SNAREs in vesicle transport [38]. Although the number of GP2 molecules required for fusion pore formation has not been reported, it is highly likely that multiple GP2 trimers are needed to promote membrane fusion based on our knowledge of influenza HA-mediated fusion[39]. The *trans*-EGP complex may provide a more efficient means than *cis*-EGP oligomers (present on virions) alone in promoting membrane hemifusion and fusion. The following observation is consistent with this hypothesis. Although evidence suggests that Ebola and Marburg viruses utilize same receptor(s) in viral entry, expression of EGP on the target cells had no enhancement effect on the MGP-mediated viral entry (see Figures 1 and S1). If *trans *enhancement did not require direct interactions between the cell-anchored and viral anchored EGPs, we would predict that EGP on the cells could enhance MGP-mediated viral entry and *vice versa*. The inability of EGP to facilitate the MGP-mediated viral entry is likely a result of sequence specificity required for *trans *enhancement. EGP and MGP share only approximately 28% amino acid identity [29]. Further, in contrast to EGP, the furin cleavage site of MGP is located within the mucin-like region, thus part of the mucin-like region resides in GP2 upon the protease cleavage [30]. Thus the sequence divergence between EGP and MGP may prevent the formation of the *trans *EGP/MGP oligomers, as proposed in Figure 5 for EGPs. In contrast, we speculate that the EGP-mediated *trans *enhancement may occur between Ebola species, since EGPs of these species share approximately 85% sequence homology. Evidence has been well documented for sequence-dictated specificity in protein-protein interactions in other systems. For example, mixed trimers between hemagglutinin proteins (HAs) derived from different influenza subtypes could not be detected experimentally because HA trimer formation is sequence specific [40]. In vesicle transport, the productive formation of the *Trans*-SNARE complex, comprising of a stable four α-helix bundle, is almost exclusively by pairing v-SNARE with its cognate t-SNARE [41]. In contrast, for class I fusion proteins of the enveloped viruses including Ebola, the six α-helix bundle is formed by homo-trimers [19,20]. Nevertheless, the formation of such oligomeric structures requires sequence specificity. Direct biochemical evidence awaits future work to demonstrate the formation of the *trans*-EGP complex, highly speculative at present, to validate the proposed model for EGP-mediated *trans *enhancement (see Figure 5). Furthermore, future studies on *trans *enhancement may provide mechanistic insights on class I fusion protein-mediated membrane fusion and viral entry.

### Dichotomous nature of Ebola GP: Interference *versus *enhancement

Previously [30] and in this study, we have shown that overexpression of EGP in the target cells specifically blocks both the EGP- and MGP-mediated viral entry (see Figure S1). However, in the current study, we also demonstrate that using a Tet-On system, expression of EGP in the target cells enhances the EGP-, but not MGP-, mediated viral entry. These seemingly paradoxical observations, we believe, are due to the dichotomous nature of EGP: Low level expression of EGP on the target cells enhances viral entry via the fusion machinery, GP2. In contrast, high level expression of EGP on the target cells can sequester the Ebola receptor(s) by RBD of GP1 and thus lead to entry interference or superinfection resistance.

In conclusion, we have demonstrated that EGP expression at low levels in target cells specifically enhances EGP-mediated transduction. The viral fusion machinery in the cell associated EGP specifically facilitates the enhancement of EGP-mediated viral transduction probably by *trans *oligomerization and cooperative interaction between virus/cell associated GP2 proteins via a mechanism analogous to vesicular membrane fusion and cell-cell fusion.

## Methods

### Cell lines and antibodies

Human embryonic kidney (293T) cells were grown in DMEM supplemented with 10% FBS, penicillin and streptomycin (100 U/ml). Human cervical carcinoma (HeLa) Tet-On cells and Human embryonic kidney (HEK) Tet-On cells were grown in DMEM with 10% tetracycline free FBS (Clontech). Human T-lymphocyte (Jurkat) Tet-On cells were grown in RPMI, 10%FBS (tetracycline free), 10 mM HEPES, 1 mM sodium pyruvate, penicillin and streptomycin (100 U/ml). Ebola GP monoclonal antibody (12B5-1-1) was kindly provided by Dr. Mary K Hart (USAMRIID). HIV p24 monoclonal antibody was obtained from AIDS Reagent Program. α-actin, goat anti-mouse-HRP and goat anti-mouse-FITC antibodies were purchased from Sigma.

### EGP expression vector construction and Mutagenesis

The Ebolavirus Zaire glycoprotein gene (EGP) and mucin deletion mutant (ΔEGP) expression vectors in pCDNA3.1 plasmid have been described previously[16]. All alanine substitution mutations of the Ebola GP gene were generated by site-directed mutagenesis with the Stratagene QuickChange^®^Site-directed mutagenesis kit according to the supplier's protocols. All mutations were confirmed by DNA sequencing the entire coding region (CRC-sequencing facility, University of Chicago). To construct inducible GP expression plasmids, Wt or mutant GP were cloned into pREV-TRE vector under a tetracycline inducible minimal CMV promoter (Clontech).

### Generation of Tet-On cell lines

HEK Tet-On cell line expressing recombinant tetracycline activator (rTA) was generated by transducing HEK cells with MLV based vector pREV-Tet-On according to manufacturer's protocol (Clontech). Transduced cells were selected with G418 at a concentration 800 μg/ml, 48 h post-transduction. EGP expressing cells were generated by transducing HEK Tet-On cells with MLV vector carrying EGP gene under tetracycline regulated promoter and selecting at 100 μg/ml concentration of hygromycin B. Similarly, HeLa Tet-On (G418 500 μg/ml; hyrgromycin B 200 μg/ml) and Jurkat Tet-On (G418 2000 μg/ml; hyrgromycin B 800 μg/ml) cells were generated. After two weeks of selection, the cells were maintained in their respective media supplemented with G418 and hyrgromycin B (100 μg/ml each).

### Analysis of EGP surface expression by flow cytometry

HEK Tet-On cells bearing Wt or mutant EGP were seeded in 12-well plates (6 × 10^4 ^cells) in 1 ml of media without G418 or hygromycin B. EGP expression was induced with different concentrations of doxycycline(0, 0.01, 0.1, 1 μg/ml). After 24 h, cells were washed once with 1 ml of PBS and dissociated from the plates using Hanks dissociation buffer (Gibco). Cells were spun at 2000 rpm and resuspended in 50 μl PBS containing 2% BSA and 1 μg of 12B5-1-1 antibody. After 30 min incubation on ice, the cells were washed twice with 300 μl of PBS containing 2%BSA. The cells were resuspended in 50 μl of PBS with 2% BSA and 0.25 μg of goat anti-mouse FITC conjugated antibody. After 20 min incubation on ice, cells were washed twice in 300 μl PBS-BSA buffer and resuspended in 100 μl PBS-BSA buffer. EGP expression levels were measured by flow cytometry, using BD FACScalibur, gating for 20,000 live cells. The data were analyzed using WinMDI 2.8 software.

### Western blot

HEK Tet-On cells were seeded in 12-well plate (3 × 10^4^) in 1 ml of media without G418 or Hyrgromycin B and EGP expression was induced with different concentrations of doxycycline(0, 0.01, 0.1, 1 μg/ml). After 48 h, the cells were lysed with 1% triton lysis buffer and analyzed by immunoblotting as described earlier[16]. Wt or mutant GP expression and incorporation were determined as previously described[16].

### Production of pseudotyped virus

GP/HIV pseudovirions for the infection assay and western blots were produced using three-plasmid based system [42]. In this system, the structural proteins (gag-pol) were provided in *trans *with the reporter gene expression vector carrying HIV RNA packaging signals (see Figure 1C). Briefly, 293T cells were co-transfected with 2.4 μg of HR'-CMV-Luc (luciferase reporter) or HR'-CMV-GFP (GFP reporter), 1 μg of Δ8.2 (gag-pol), and 0.5 μg of EGP or MGP or VSV-G plasmids using lipofecatamine™2000.

### Infection assay in GP expressing Tet-On cells

HEK Tet-On cells were seeded in 24-well plates at a density of 3 × 10^4^cells/well in 0.5 ml media and induced with varying concentration of doxycycline (0, 0.01, 0.1, 1 mg/ml). After 24 h, without removing the old media, cells were infected with 0.5 ml of HIV pseudovirions carrying luciferease or GFP reporter gene. The media was replaced 12 h post-infection. The luciferase activities were measured 48 h post-infection as an indirect measure of GP mediated viral entry, and presented as percent infectivity of the uninduced cells. In the case of GFP reporter virus, infected were observed under a fluorescent microscope 72 h postinfection and percentage of infected cells quantified using BD FACScalibur.



### VLP-based fusion assay

#### (1) Construction of VP40 expression vectors

The Zaire Ebola VP40 gene was synthesized by multiple rounds of overlapping PCR based on EBOZ genome sequence (Gene accession number L11365). β-lactamase gene was PCR amplified from pCDNA3.1 vector and fused to N-terminal of VP40 to create a modified β-lactamase-VP40 fusion protein (BlaM-VP40) by a linker sequence (GSGGGSGGT). The modified β-lactamase lacks the N-terminal 24 amino acids and His24 was substituted by Asp to create an optimal Kozak sequence (Invitrogen).

#### (2) Production of VLPs

Briefly, 293T cells were co-transfected with 3 μg Bla-VP40 and 0.5 μg glycoprotein plasmid (EGP or MGP or VSV-G or EnvA) using lipofecatamine™2000. The supernatant containing the VLPs were collected twice (24 h and 48 h post-transfection), combined and clarified of floating cell debris by centrifugation at 3,000 rpm for 10 min.

#### (3) VLP infection assay

Target cells were seeded in 24-well plates at a density of 2 × 10^5^cells/well in 0.5 ml media. After 24 h, 0.5 ml of viral supernatant were added to each and spinoculated at 1,500 rpm for 2 h (4°C). After 2 h, the plates were incubated at 37°C for another 3 h. The cells were washed once with HBSS to remove unbound virus and infected cells were detected by using LiveBLAzer -FRET B/G substrate according to manufacturer's recommendations (Invitrogen). The infected cells were visualized by Olympus XI70 microscope or quantified by flow cytometry using a CyAn™ ADP (Dakocytomation).

## Competing interests

The authors declare that they have no competing interests.

## Authors' contributions

BM performed the experiments, BM and LR designed the experiments and drafted the manuscript.

## Supplementary Material

Additional file 1 **Experimental Procedures**. Experimental protocols for the additional files. Click here for file

Additional file 2 **Supplementary figure legends**. Description of the additional figures. Click here for file

Additional file 3 **Fig. S1**. Over-expression of ΔEGP blocks EGP/and MGP/HIV entry. Click here for file

Additional file 4 **Fig. S2**. Cell surface expression of EGP in Tet-On cells. Click here for file

Additional file 5 **Fig.S3**. Western blot analysis of EGP expression in Tet-On cells. Click here for file

Additional file 6 **Fig. S4**. Analysis of EGP mutants. Click here for file
