# Supplementary material for: Expression of Ebolavirus glycoprotein on the target cells enhances viral entry
Source: Virol J. 2009 Jun 8;6:75. doi: 10.1186/1743-422X-6-75 (PMC2699336; doi:10.1186/1743-422X-6-75)
Supplement: Additional file 1 — Experimental Procedures. Experimental protocols for the additional files. [file 1743-422X-6-75-S1.doc]

**Supplementary- Experimental Procedures**

**Entry interference assay**

**Entry interference assay was performed following a previous protocol developed by us with modifications. Briefly, one day prior to transfection, 8X10^5^ 293T cells were seeded in 6-well plates coated with poly-L-lysine (Sigma). The next day cell were transfected with varying amounts (0, 1, 4 μg) of expression plasmid (ΔEGP or EnvA) using lipofectamine 2000 following supplier’s protocol . In all transfections, the total amount of DNA was kept at 4μg by adding the control vector (pCDNA3.1). After 5h, the transfection mix was replaced with 3 ml fresh media. At 60h post-transfection, the cells were washed once with PBS and dissociated from the plate using Hank based cell dissociation buffer (Gibco). Approximately 1X10^5^ cells in 0.2 ml of fresh media were seeded into 48-well plates. Immediately 0.2ml of pseudotyped viruses (HR’-CMV-Luc or HR’-CMV-GFP) was added to each well and mixed.** Eight hours p.i., the supernatant was removed and 0.4ml of fresh media was added**.** The cells were lysed in 100 µl of cell culture lysis reagent (Promega) at 48 h p.i. The luciferase activities of the target cells were measured with a luciferase assay kit (Promega) and an FB12 luminometer (Berthold detection system) according to the supplier's protocol. The luciferase activity in each sample was measured in triplicates. Experiments were repeated three times or more times.
