# Supplementary material for: Expression of Ebolavirus glycoprotein on the target cells enhances viral entry
Source: Virol J. 2009 Jun 8;6:75. doi: 10.1186/1743-422X-6-75 (PMC2699336; doi:10.1186/1743-422X-6-75)
Supplement: Additional file 2 — Supplementary figure legends. Description of the additional figures. [file 1743-422X-6-75-S2.doc]

**Supplementary- Figure legends**

**Figure S1. Over-expression of ΔEGP blocks EGP/ and MGP/HIV entry.** 293T cells were transfected with ΔEGP or EnvA expression plasmids. After 48h, the cells were challenged with EGP/HIV, MGP/HIV or VSV-G/HIV pseudovirions carrying a luciferase reporter gene. The luciferase activities in the cell lysates were measured 48h post-infection and are presented as percentage of the luciferase activity in control vector transfected cells.

(A).Pseudotyped virus entry in cells expressing ΔEGP (left panel). Pseudotyped virus entry in cells expressing EnvA, the envelope glycoprotein of ASLV-A (right panel).

(B). Expression of ΔEGP in 293T cells by flow cytometry using a GP monoclonal antibody.

**Figure S2**. Cell surface expression of EGP in Tet-On cells. Wt or mutant EGP Tet-On cells were seeded in 12-well plates (6X10^4^ cells/well) and EGP expression was induced with indicated concentrations of dox. After 24h post-induction, cell surface EGP levels were analyzed by flow cytometry using a GP monoclonal antibody.

**Figure S3.** Western blot analysis of EGP expression in Tet-On cells. Wt or mutant EGP Tet-On cells were seeded in 12-well plates and induced with indicated concentrations of dox. Forty-eight hours post-induction, cell lysates were subjected to SDS-PAGE followed by immunoblotting using a EGP monoclonal antibody.

**Figure S4. Analysis of EGP mutants**.

1. Western blot analysis of GP incorporation levels in HIV particles. Top panel: Incorporation level of EGP in HIV particles detected using an EGP1 monoclonal antibody. Bottom panel: HIV p24 levels were detected using a anti-p24 monoclonal antibody and were used as loading controls.
2. Western blot analysis of EGP expression in 293T cells. Top panel: EGP expression levels in 293T cells transfected with EGP expression plasmids were detected using an EGP1 monoclonal antibody. Bottom panel: a-actin levels in cell lysates were detected using an anti-actin monoclonal antibody and were used as a loading control.
