# Supplementary figures and images for: Expression of Ebolavirus glycoprotein on the target cells enhances viral entry
Source: Virol J. 2009 Jun 8;6:75. doi: 10.1186/1743-422X-6-75 (PMC2699336; doi:10.1186/1743-422X-6-75)

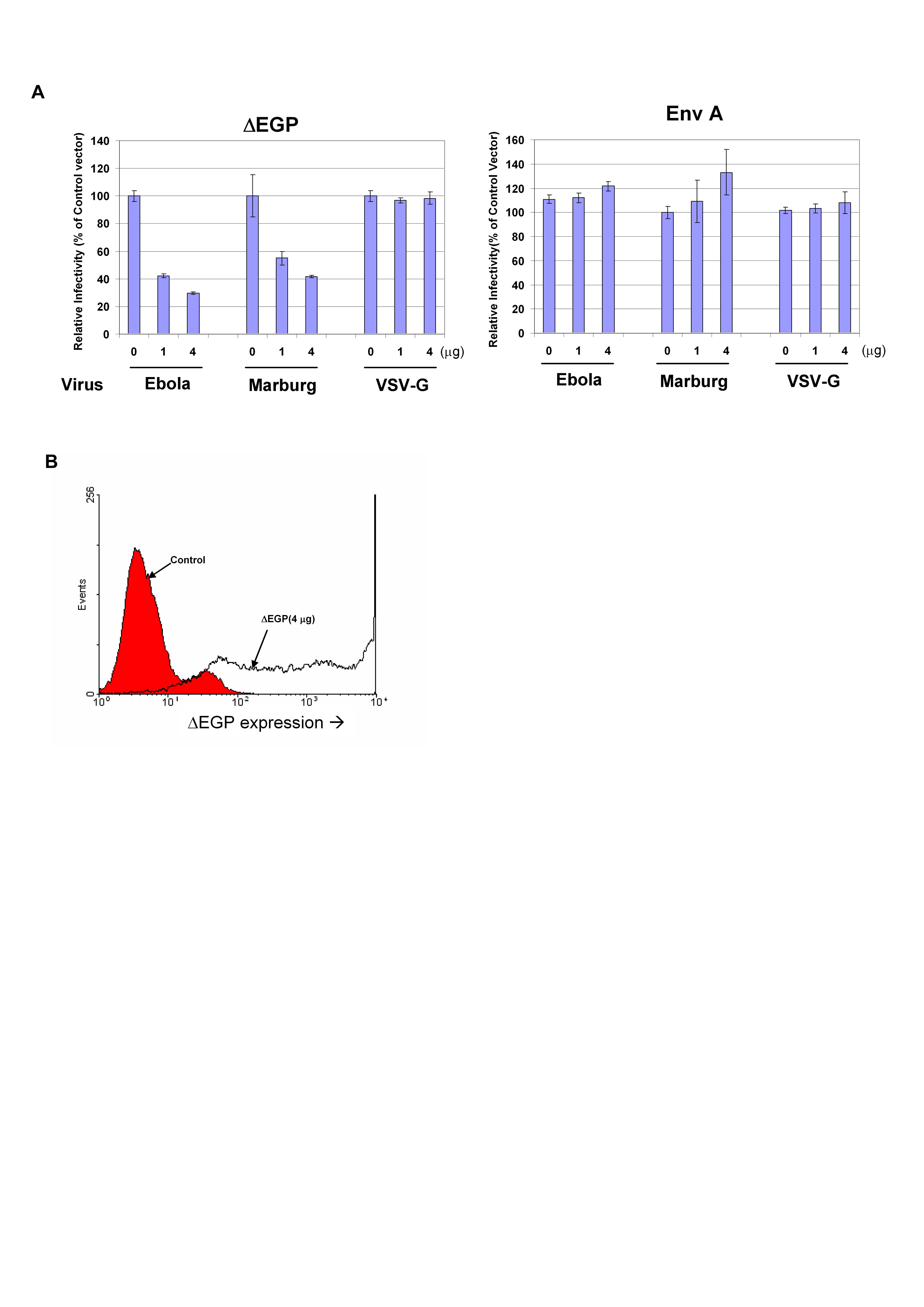

Supplement: Additional file 3 — Fig. S1. Over-expression of ΔEGP blocks EGP/and MGP/HIV entry. [file 1743-422X-6-75-S3.jpeg]

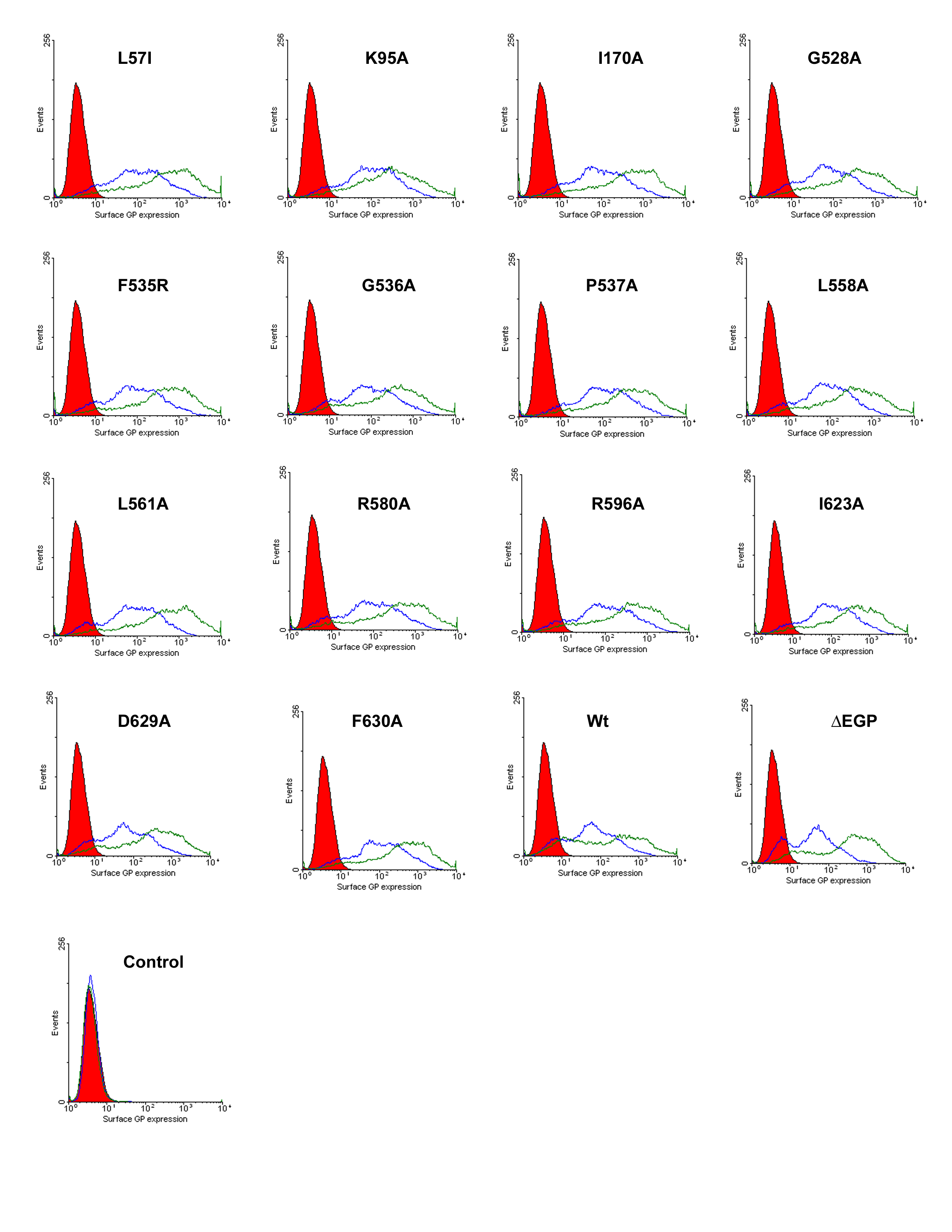

Supplement: Additional file 4 — Fig. S2. Cell surface expression of EGP in Tet-On cells. [file 1743-422X-6-75-S4.bmp]

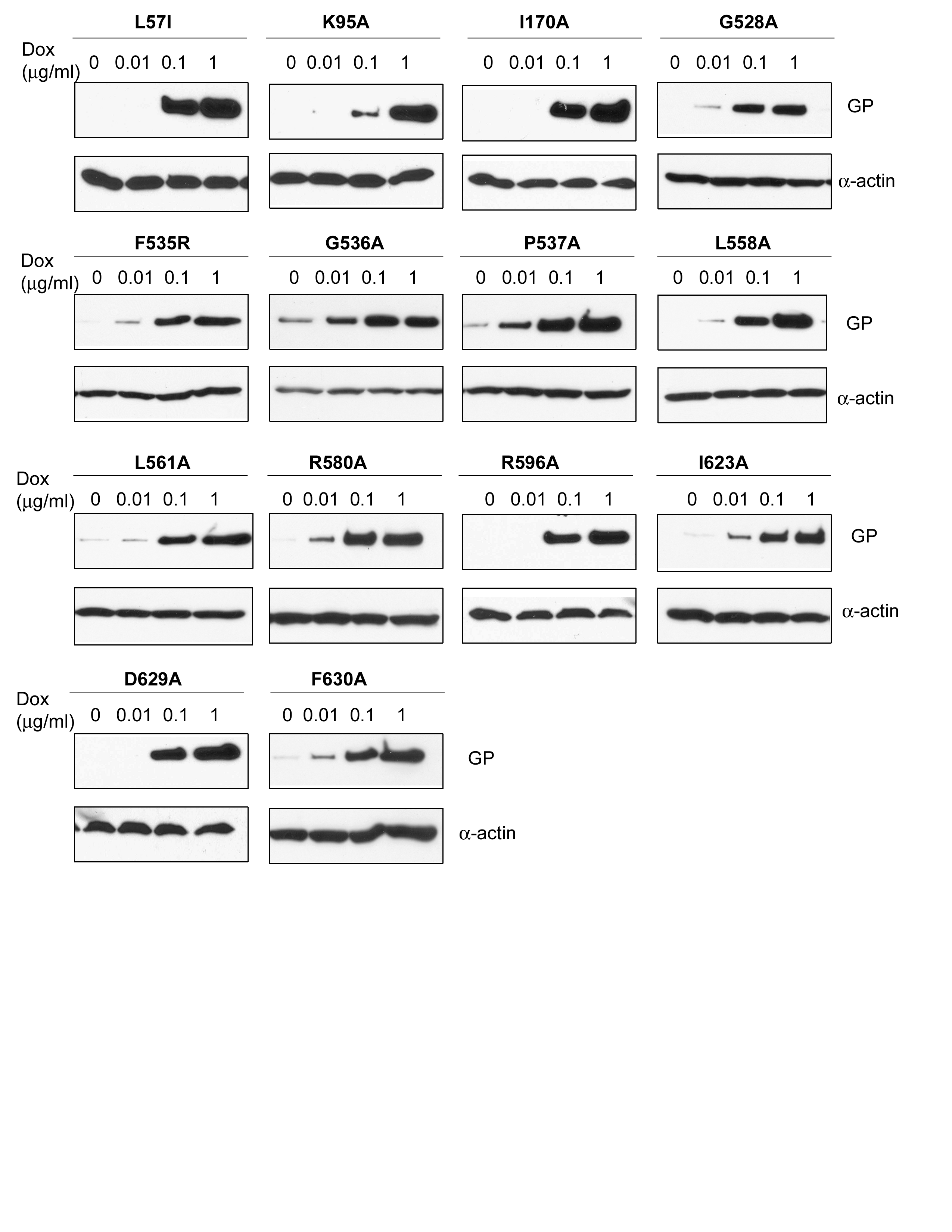

Supplement: Additional file 5 — Fig.S3. Western blot analysis of EGP expression in Tet-On cells. [file 1743-422X-6-75-S5.tiff]

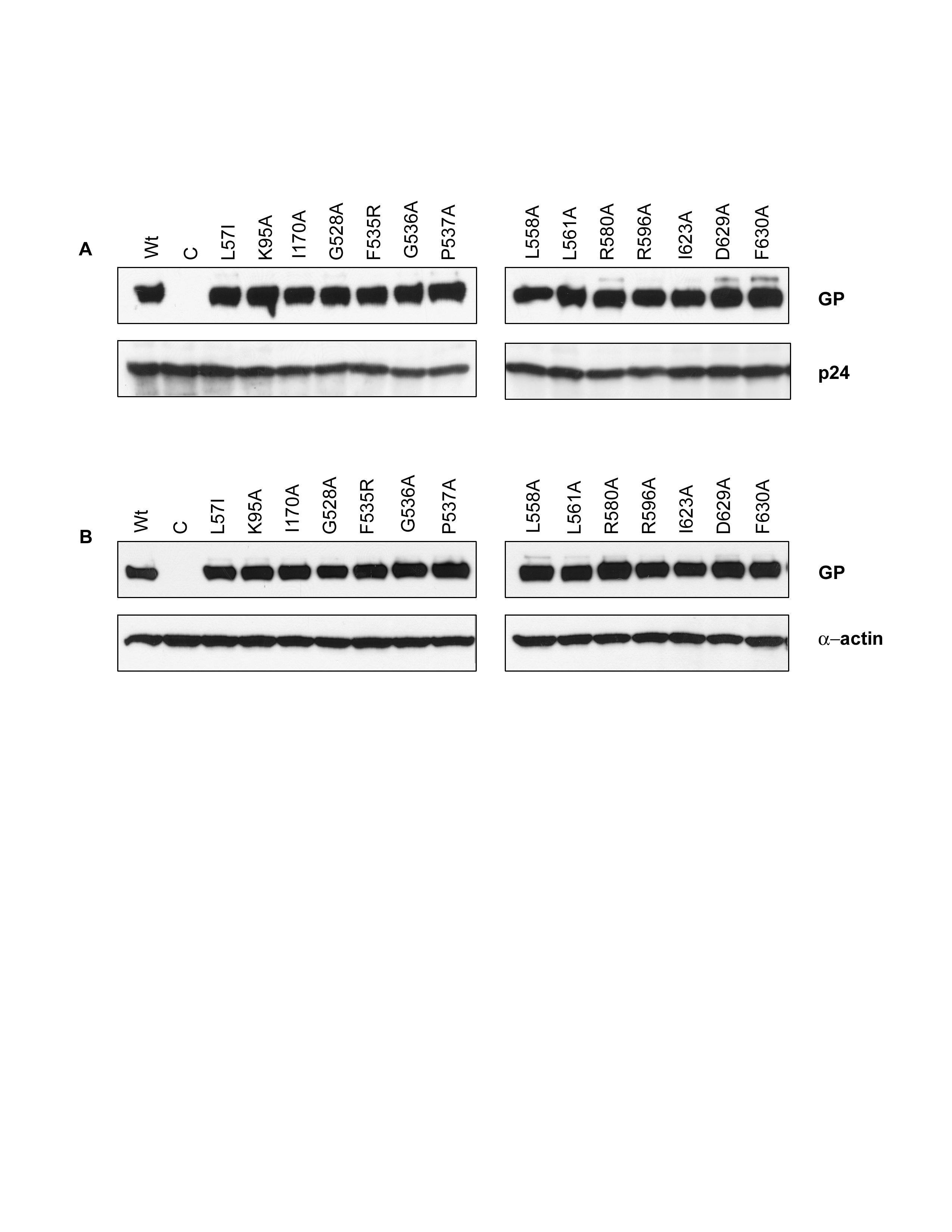

Supplement: Additional file 6 — Fig. S4. Analysis of EGP mutants. [file 1743-422X-6-75-S6.tiff]
